# Chronic Stress, Immune Suppression, and Cancer Occurrence: Unveiling the Connection Using Survey Data and Predictive Models

**DOI:** 10.3390/medsci14020245

**Published:** 2026-05-08

**Authors:** Teddy Lazebnik, Vered Aharonson

**Affiliations:** 1 Department of Information Science, University of Haifa, Haifa 3498838, Israel; 2 Department of Computing, Jonkoping University, 553 18 Jonkoping, Sweden; 3 Department of Basic and Clinical Sciences, Medical School, University of Nicosia, 2414 Nicosia, Cyprus; aharonson.v@unic.ac.cy; 4 School of Electrican and Information Engineering, University of the Witwatersrand, Johannesburg 2050, South Africa

**Keywords:** survey-based modeling, computational psychosocial oncology, predictive health, socio-demographic personalized medicine

## Abstract

Chronic stress has been implicated in cancer occurrence, but a direct causal connection has not been consistently established. Machine learning and causal modeling offer opportunities to explore complex causal interactions between psychological chronic stress and cancer occurrences. We developed predictive models employing variables from stress indicators, cancer history, and demographic data from self-reported surveys to examine the direct association between chronic stress and cancer occurrence, as well as a hypothesized immune-suppression-mediated pathway. The models were corroborated by traditional statistical methods. Our findings indicated significant associations between stress frequency, stress level, perceived health impact, and cancer occurrence. Although stress alone showed limited predictive power, integrating socio-demographic and familial cancer history data significantly enhanced model accuracy. These results highlight the multidimensional nature of cancer risk, with stress emerging as a notable factor alongside genetic predisposition. These findings strengthen the case for addressing chronic stress as a modifiable cancer risk factor, supporting its integration into personalized prevention strategies and public health interventions to reduce cancer incidence.

## 1. Introduction

Cancer remains a leading cause of mortality worldwide [1]. Its occurrence and development are influenced by a complex interplay of genetic [2], environmental [3], and physiological factors [4]. Psychological stress is increasingly being considered a factor in cancer risk [1,5], and in the inhibition of cancer treatment efficacy [6,7]. Chronic stress can disrupt homeostatic processes, leading to sustained hormonal and inflammatory responses [8]. These processes weaken the immune system, which is vital for defending against tumor development [9].

While physiological studies and animal models have provided insights into possible mechanisms by which chronic stress can induce cancer, epidemiological human studies have portrayed inconsistent results regarding this association [10]. An empirical validation of the stress–cancer link in human populations remains underexplored. Namely, currently, there is no clear evidence that chronic stress causes cancer occurrence either directly or through immune suppression.

Machine learning (ML) has emerged as a powerful tool for the analysis of complex, multi-dimensional health data, where it can uncover hidden patterns and support accurate prediction [11]. ML models have been successfully applied to cancer risk prediction [12,13], psychological health assessment [14,15], and immune response modeling [16,17]. Nevertheless, predictive ML and causal inference serve different scientific purposes. Predictive ML is designed to detect complex associations and improve out-of-sample prediction, whereas causal analysis is required to test whether chronic stress has a direct effect on cancer occurrence or an indirect effect through immune suppression. To the best of our knowledge, these two objectives have not yet been integrated in a single survey-based framework for studying the link between chronic stress, immune suppression, and cancer occurrence.

In this study, we pursue two complementary goals using self-reported survey data. First, we use ML methods to identify predictive patterns and complex associations between stress perception measures, immune suppression indicators, socio-demographic variables, family cancer history, and cancer occurrence. This component of the study is intended for prediction and pattern discovery rather than causal interpretation. Second, we use statistical analysis and the Rubin Causal Model to test the hypothesis that chronic stress is associated with cancer occurrence, including a potential immune-mediated pathway through immune suppression. Accordingly, causal conclusions in this study rely on the statistical and causal analyses, whereas the ML models are used to evaluate predictive utility and to characterize the multidimensional structure of the data. This structured design allows us to distinguish between predictive performance and etiological interpretation, and to examine both questions without conflating association, prediction, and causation. Using these models, we investigate a possible direct association between chronic stress and cancer occurrence, alongside a hypothesized immune-suppression-mediated pathway.

The reminder of the manuscript is organized as follows. Section 2 reviews the known factors between chronic stress and cancer progression, stress-induced immune suppression, and immune suppression influence on cancer emergence. Section 3 presents the survey conducted, machine learning modeling, and the statistical analysis conducted. Section 4 outlines the obtained results. Finally, Section 5 discusses the obtained results in their socio-clinical context and suggests future work.

## 2. Related Work

In this section, we examine the interrelationships between chronic stress, immune suppression, and cancer occurrence, addressing each pair of factors independently.

### 2.1. Chronic Stress and Cancer Occurrence

The associations between severe life events, anxiety, depression, and insufficient social support with increased cancer occurrence have been extensively reviewed in the past two decades [18,19]. Stress management strategies, including pharmacological, physical, social, and psychological approaches, could be instrumental in cancer prevention efforts [20]. Recent epidemiological evidence suggests that chronic psychological stress may be a risk factor for cancer, particularly for breast, colorectal, lung, prostate, and pancreatic cancers [7,21,22,23,24]. However, results from both epidemiological and clinical studies were inconsistent [10,25,26]. Moreover, the studies reveal that methodological challenges in assessing the impact of stress on cancer occurrence may hamper the identification of this association [27]. For instance, they may not elicit the contribution of smoking, alcohol consumption, and poor diet, which may be stress-induced lifestyle behaviors, to cancer occurrence [7,28]. The reliable measurement of chronic stress is another significant challenge of these population-based studies [29]. Finally, the mechanisms by which chronic stress influences cancer are complex and not yet fully understood. Prolific research focused on elucidating the molecular mechanisms through which chronic stress facilitates tumor occurrence and progression, with immune suppression being identified as a key mechanism [30].

### 2.2. Chronic Stress and Immune Suppression

Chronic stress has been consistently linked to the suppression of immune function [30,31,32], of both cellular and humoral immunity [33]. Recent research has highlighted mechanisms by which chronic psychological stress can modulate immune function. These include pathways involving the hypothalamic–pituitary–adrenal axis and the sympathetic nervous system [18,30,34,35]. Stress-related elevations in cortisol and catecholamines can suppress immune surveillance by reducing the activity of natural killer cells, impairing T-cell function, and altering cytokine profiles [36,37,38,39]. Animal studies further demonstrated that chronic stress impairs T cell function through decreased T cell proliferation, altered cytokine secretion, and increased lymphocyte apoptosis [40,41,42]. Inducing low-grade chronic inflammation, this dysregulation increases proinflammatory factors, and suppresses the numbers, trafficking, and function of immunoprotective cells [43,44]. These changes may foster an environment conducive to tumor initiation and progression [45]. The autonomic nervous system, activated by stressors like anxiety, pain, sleep disorders, or depression, is considered to play a crucial role in this process [46,47,48]. The dysregulation impact on both innate and adaptive immunity was associated with attenuated vaccine responses, impaired control of latent viruses, and heightened inflammation in patients [18,49]. Decreased activity of tumor-infiltrating T lymphocytes was associated with higher levels of stress in ovarian cancer patients [38,50], and with increased susceptibility to skin cancer [51].

The studies linking stress to immune suppression convey multiple limitations. A gap persists in the translation of laboratory findings to clinical applications. The individual differences in stress tolerance may limit the generalizability of results. The underlying mechanisms and directionality of associations between variables such as age, gender, socio-economic status, and stress-induced immune suppression are not yet well-understood. Understanding these mechanisms is crucial for developing strategies that could mitigate the adverse effects of stress on immune function and overall health. Finally, a direct link between these immune alterations and disease outcomes is often absent in studies.

### 2.3. Immune Suppression and Cancer Occurrence

Psychoneuroimmunology research has highlighted the significant role of immune function in tumor behaviors [52,53]. Immunosuppression, whether primary, secondary, or induced by cancer itself, is associated with increased cancer incidence, particularly of skin cancers, and often exhibits patterns observed during fetal development [54,55,56]. Targeting tumor-induced immune suppression—encompassing regulatory cells, cytokines/chemokines, T cell exhaustion, and metabolic factors—is a promising approach for improving cancer immunotherapy [57,58]. However, while evidence underscores that certain aggressive cancers tend to develop in immunocompromised hosts, the factors influencing this, including specific aspects of immune deficiency and variations in patient populations, remain underexplored [59].

Taken together, the current literature suggests potential links between chronic stress, immune suppression, and cancer occurrence. The studies employ diverse methodologies for assessing stress and include various study populations, which can impact the generalization of the findings. There remains a lack of sufficient data to conclusively determine the influence of gender, age, and socio-economic status on these connections. Further research is necessary to explore how these relationships may differ across populations and to identify underlying mechanisms. Such research would benefit from the adoption of standardized stress measurement tools and the concurrent analysis of multiple moderating factors. Understanding the relationship between chronic stress, immune suppression, and cancer risk may allow for early identification of at-risk populations. Harnessing this understanding for the development of targeted prevention strategies that address stress-related modifiable risk factors may reduce cancer incidence [7].

## 3. Methods and Materials

**Figure 1:**
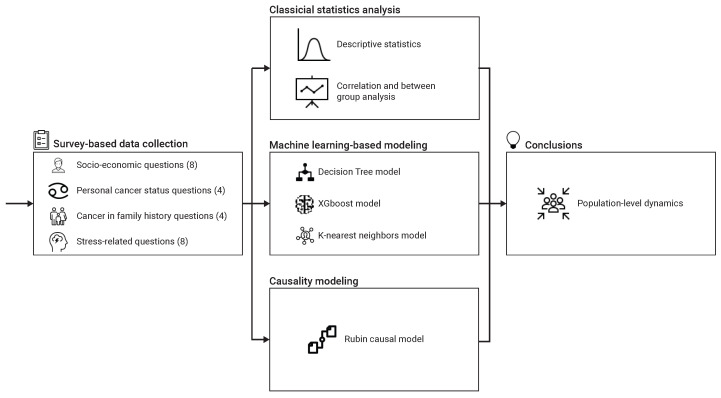
A schematic view of the methodological flow of this study.

We employed a three-phased workflow, outlined in Figure 1. Initially, we developed a four-section survey to gather data (The full survey is provided as Appendix A). Next, we analyzed the data using three complementary methods: a descriptive statistics and linear regression model, non-linear machine learning-based regression models, and causal modeling. Based on the combined results, we interpreted potential population-level dynamics.

### 3.1. Survey

To gather real-world data on stress and cancer status, we conducted a questionnaire consisting of four sections. The first section includes eight questions on the participant’s socio-demographic and economic background ($q_{1}^{1}-q_{8}^{1}$): country, gender, age, marital status, number of children, education, income, and household size. These factors are used in two contexts: (1) a validation that the sample is broad and representative; and (2) an investigation of the effect of socio-economic factors on the relation between stress and cancer occurrence. These factors were picked based on their documented importance for healthcare and social policy design [60,61,62,63,64]. The second section includes four questions on the participant’s cancer status, focusing on personal experiences with the disease ($q_{1}^{2}-q_{4}^{2}$): whether the participant has had cancer, and if so, what type, when, and whether the participant is still undergoing cancer treatment [65,66]. These questions are used for the model parameter estimation, where cancer status is the response variable. The third section includes four questions on the family’s cancer status, focusing on the participant’s immediate family experience with the disease ($q_{1}^{3}-q_{4}^{3}$): has one of the participant’s immediate family members had cancer, and if so, what type and what is their relation to the participant; and how many immediate family members have been diagnosed with cancer. These questions are used to control for genetically related cancer properties [67,68,69]. The survey was intentionally designed to focus on stress-related, socio-demographic, and family-history domains. Accordingly, it did not include several established cancer risk factors, such as smoking, alcohol consumption, diet, body mass index, physical activity, comorbidities, and environmental or occupational exposures. This design makes the study suitable for exploratory analysis of stress-related patterns. The fourth section includes eight questions ($q_{1}^{4}-q_{8}^{4}$) on the participant’s stress and physiological stress-related symptoms: daily-life stress, a belief that stress affects physical health, coping with stress, sleep difficulties, loss of appetite, fatigue, trouble focusing, and the impact of stress on mental health. Four of these items were used to estimate a general self-reported stress score, whereas the remaining four items were used as an exploratory proxy for stress-related physiological dysregulation, which we refer to in the manuscript as immune suppression [70,71,72,73]. This operationalization was not intended to serve as a direct clinical measurement of immune function, but rather as a survey-based population-level proxy in a setting where biomarker data were not available. All questions in the stress section used a Likert scale [74] from one to five, indicating the least stress to the most stress, respectively.

Responses to the questionnaire were gathered using an online survey distributed across multiple channels, including email, social media, and community outreach programs. The questionnaire was designed to be inclusive, capturing responses from individuals across diverse socio-economic backgrounds. Participation was voluntary, with respondents informed about confidentiality and ethical considerations. Data collection was facilitated through Google Forms [75], which automatically anonymized responses to ensure privacy and adherence to ethical standards. The questionnaire remained open for four months, from December 2024 to March 2025.

Because the study relied on a self-reported survey, particular attention was given to the design of the questionnaire in order to improve construct validity and reduce common method bias [76]. Specifically, the questionnaire was organized into multiple sections representing distinct constructs, including socio-demographic characteristics, personal and family cancer history, stress perception, and immune-related indicators. In addition, the survey combined different question types, including Likert-scale items, binary questions, categorical responses, and open-ended cancer-type items, thereby reducing the uniformity of response patterns across variables. Several key variables, such as personal cancer diagnosis and family cancer history, were also formulated as relatively objective self-reports rather than attitudinal judgments, which may reduce susceptibility to shared subjective response tendencies. Moreover, the questionnaire items were selected on the basis of the prior literature and established epidemiological considerations, supporting the content validity of the measured domains.

Furthermore, since the survey was distributed through open online channels rather than through a closed registry or a pre-defined roster, the sampling frame could not be fully specified and a response rate could not be calculated. This recruitment strategy enabled broad and geographically diverse participation, but it may also have introduced selection bias by preferentially including individuals with internet access, higher digital literacy, and greater willingness to participate in health-related surveys. In addition, as the questionnaire relied on self-reported information about stress, health, and cancer-related experiences, responses may have been affected by social desirability and recall biases. To reduce these risks, participation was voluntary and anonymous, no identifying information was collected, confidentiality was explicitly stated before participation, and the survey was disseminated across multiple channels to increase heterogeneity in the sample. Nevertheless, these procedures can mitigate but not eliminate recruitment and reporting biases.

### 3.2. Analysis Methods

We conduct three types of analysis that examine the dynamics in the data collected from the survey: statistical analysis, ML modeling, and causality modeling. Each of the analyses aims to capture a different property of the collected data. The statistical analysis aims to provide relatively simple yet straightforward descriptive statistics with correlations in the data and between-group analysis. The ML modeling ranges from a computationally simple (Decision Tree) and explainable model to a computationally complex (XGBoost) and unexplainable model to study non-linear connections in the data. Finally, the Rubin causal modeling analysis aims to estimate assumption-dependent effects and structured associations between stress, immunity suppression, and cancer occurrence. For the analysis, We use Python (version 3.11) [77]. The *p*-value for statistical significance was set at p≤0.05.

#### 3.2.1. Conceptual Variables Definitions

We define the conceptual variables used in this study based on the survey questions. Formally, the cancer occurrence variable is binary and is based on the question “Have you ever been diagnosed with cancer?” in the survey. To obtain a single stress variable, we average the four stress questions in the survey’s fourth section. This stress-level average is standardized to a value from 0 to 1 by subtracting one from the value and dividing it by five [74]. The other four questions from the fourth section of the survey (sleep difficulties, loss of appetite, fatigue, and trouble focusing) are used as a normalized self-reported proxy for stress-related physiological dysregulation, which we denote as immune suppression for brevity. This variable should be interpreted cautiously, as it does not directly measure biological immune function, but rather captures symptoms that may accompany chronic stress-related dysregulation in large-scale survey settings where objective biomarkers are unavailable. All other questions in the survey are treated as individual variables in order to allow us to investigate the influence of each of these on the stress and cancer relationship, with or without immunity suppression.

#### 3.2.2. Statistical Analysis

Three types of descriptive statistics are included in our analysis. Correlation analysis involving stress-related variables was performed using the Spearman rank correlation coefficient, which is appropriate for ordinal or non-normally distributed data [78]. Associations involving the binary cancer occurrence variable were evaluated using the $\chi^{2}$ test [79]. Between-group comparisons were performed using either Student’s *t*-test or an Analysis Of Variance (ANOVA) with post hoc t-tests and Bonferroni correction [80,81]. Regression analysis was performed by a logistic model utilizing the least mean squares method [82,83]. The analysis results are presented as mean standard deviation (SD) for the continuous variables and as percentages for the categorical variables. To assess the robustness of the logistic regression model, we evaluated multicollinearity among the independent variables using the Variance Inflation Factor (VIF) [84]. VIF values below commonly accepted thresholds were interpreted as indicating no substantial multicollinearity. In addition, model calibration and goodness-of-fit were assessed using the Hosmer–Lemeshow test [85], where a non-significant result was interpreted as evidence of an adequate fit between predicted and observed outcomes.

In order to further assess the potential influence of common method bias resulting from the use of a single self-report instrument, we conducted Harman’s single-factor test [86] on the main survey-derived variables. The first unrotated factor accounted for 14% of the total variance, suggesting that common method variance was unlikely to fully account for the observed associations. In addition, the analytical framework itself partially mitigates this concern, as the models combine heterogeneous predictor types, including socio-demographic variables, family cancer history, and stress-related measures, rather than relying on a single latent self-reported construct. Finally, in the causal analysis, propensity score matching was used to balance observed covariates between treatment conditions, thereby reducing the risk that the estimated treatment effects were driven solely by systematic differences in self-reported participant characteristics.

#### 3.2.3. Machine Learning Modeling

We employed three machine learning models: Decision Trees (DTs) [87], XGBoost [88], and k-Nearest Neighbors (KNNs) [89]. Each model offers distinct benefits for the understanding and prediction of relationships in the dataset. The DT model offers interpretability and the ability to handle non-linearity while maintaining clear decision boundaries [90,91]. XGBoost can capture complex interactions within the data, but is not explainable [92]. The KNN model can predict an outcome—in our case, a cancer occurrence—for an individual, using the similarity of this individual to other individuals. To leverage this property, the KNN model is trained both on the entire variable set and on three sets of variables—only socio-demographic, only stress, and stress combined with socio-demographic—and the model’s performance was compared.

Prior to model training, the dataset was preprocessed to ensure compatibility with the different ML algorithms and to reduce the risk of biased estimates due to incomplete data. Due to the data collection process, which does not allow for submitting the survey without answering all questions, no missing values were found in the dataset. In addition, categorical variables were transformed using one-hot encoding. Furthermore, feature scaling was applied when required by the model type. Specifically, continuous variables were standardized using *z*-score normalization for the KNN model, which is distance-based and sensitive to scale differences between variables. In contrast, the tree-based models (DT and XGBoost) were trained on unscaled features, as these models are generally invariant to monotonic transformations of variable scale. Importantly, all preprocessing operations were fitted using only the training data within each training–validation split and then applied to the corresponding validation or test data, in order to avoid data leakage.

We divided the dataset into a training set and a testing set that consisted of 80% and 20% of the data, respectively. This division ensures that the models have ample training data to learn from, while the separate test set allows for performance evaluation and assessment of generalization to new data [93]. We employed five-fold cross-validation to tune the models. Notably, within each fold, preprocessing parameters were estimated exclusively on the training partition and were not informed by the held-out validation partition. This technique entails a split of the training data to five subsets. Each model is trained on four subsets and validated on the fifth. The process is repeated five times so that each subset serves as a validation set once [94]. This technique mitigates issues related to variability in training data and ensures reliable performance metrics [95]. Importantly, to ensure robust evaluation in clinical ML, the dataset was divided into training and validation cohorts with statistically similar age and sex distributions. This ensures the validation set reflects the training data. In our five-fold cross-validation, all five subsets needed to maintain similar age and sex distributions [96]. The dataset was divided into five equal and distinct subsets such that the average distributional difference in age and sex between any two subsets is minimized. This optimization problem is conceptually related to the NP-hard nurse scheduling problem [97,98]. We achieved a near-optimal solution using the Directed Bee Colony Optimization algorithm [99,100]. A grid search [101] for the optimal hyperparameters of each model was conducted. The grid search systematically evaluates combinations of hyperparameters to find the configuration that yields the best performance. The hyperparameters were the depth and minimum samples per split for the Decision Trees model [102], the learning rate, maximum depth, and the number of trees for the XGBoost model [103], and the number of neighbors and choice of distance metric (e.g., Euclidean or Manhattan distance) for the KNN model [104].

The trained models were evaluated on the test set using standard classification metrics: accuracy, precision, recall, and $F_{1}$ score [105,106]. To interpret the models and understand the contributions of different features, we employed feature importance [107,108,109] and Shapley Additive Explanations (SHAP) analysis [110,111]. The feature importance was implemented using the variable permutation method that evaluates the significance of each feature by randomly shuffling its values and measuring the corresponding drop in model performance. Features causing a greater reduction in performance are considered more important. The SHAP values provide a complementary interpretable measure of the impact of the variable by estimating each variable’s contribution to the model’s predictions. SHAP assigns positive or negative values to each feature, indicating whether they increase or decrease the likelihood of a particular outcome.

#### 3.2.4. Causal Modeling

To establish causal relationships between stress, immunity suppression, and cancer occurrence, we implement the Rubin Causal Model (RCM) [112]. The RCM framework estimates treatment effects while accounting for potential confounders. All theoretically motivated survey variables were retained as prespecified covariates in the multivariable and causal analyses in order to ensure consistent adjustment across models and enable comparison between modeling frameworks, regardless of whether a given variable reached statistical significance in an individual analysis. The fundamental concept of RCM is a comparison between potential outcomes: the outcome if an individual receives a treatment versus the outcome if they do not. In our context, the “treatment” is defined as experiencing high-stress levels, and the outcome is cancer occurrence. Importantly, due to the fact that the present data are cross-sectional, temporal ordering between exposure, mediator, and outcome cannot be fully established; therefore, the resulting estimates should be interpreted as model-based estimated effects under assumptions, rather than definitive causal effects.

The formal definition of the model is as follows. Let $Y_{i}\left(1\right)$ and $Y_{i}\left(0\right)$ represent the outcomes for individual *i* under treatment and control conditions, respectively. The treatment effect for individual *i* is defined as $\tau_{i}:=Y_{i}\left(1\right)-Y_{i}\left(0\right)$. Since we can only observe one of these outcomes per individual, we estimate the outcome by the average treatment effect (ATE) ATE:=E[Y(1)]−E[Y(0)]. To mitigate selection bias, we employ propensity score matching (PSM) [113]. The propensity score e(X) represents the probability of an individual receiving treatment given their observed covariates *X* such that e(X):=P(T=1|X). We estimate e(X) using the logistic regression model, ensuring that treatment and control groups are balanced in terms of covariates. Matching is then performed to compare treated and untreated individuals with similar propensity scores. In practice, propensity scores were estimated using a logistic regression model in which the high-stress exposure indicator was regressed on the prespecified covariates included in the multivariable analysis. Participants in the exposed and unexposed groups were then matched using nearest-neighbor matching at a ratio of 1:1, without replacement. Matching was restricted to observations within the region of common support. After matching, covariate balance was assessed using standardized mean differences for the matched variables, and values below the conventional threshold were interpreted as indicating adequate balance between the groups. After adjusting for observed confounders using PSM, we estimated the effect under the modeling assumptions using regression models. Formally, we compare the adjusted means of the treatment group to the control group to derive causal conclusions.

## 4. Results

### 4.1. Statistical Analysis Results

This section includes three parts: descriptive statistics of the sample, followed by the results of the bivariate analysis, and then the multivariate analysis results.

A cohort of 1318 participants responded to the questionnaire, comprising 745 females (56.5%), 566 males (43.0%), and seven individuals (0.5%) who preferred not to disclose their gender. The participants’ age range was 18 to 79 years, with a mean of 35.7 years and a standard deviation of 13.4. Most participants (820, 62.2%) were married, 283 (21.5%) were in a relationship, 214 (16.2%) were single, 105 (8.0%) were divorced, and 48 (3.6%) were widow/ers. Most of the participants had two children (515, 39.1%) followed by the group of participants with no children (363, 27.5%), one child (158, 12,0%), three (101, 7.6%), four (93, 7.0%), and five or more (88, 6.6%) children. Regarding education, 89 (6.8%) participants did not complete high school, 267 (20.3%) completed high school, 426 (32.3%) held a Bachelor’s degree, 368 (27.9%) held a Master’s degree, and 168 (12.7%) held a PhD or M.D. Participants reported living with a median of three other individuals in their household, with 231 (17.5%) living alone, 329 (25.0%) living with one other person, 416 (31.5%) living with two others, 227 (17.2%) living with three others, and 115 (8.7%) living with four or more cohabitants. In terms of household income, 198 participants (15.0%) reported earning up to 2150 USD per month. A total of 134 participants (10.2%) reported incomes between 2150 and 2700 USD, 143 (10.8%) between 2700 and 3250 USD, 239 (18.1%) between 3250 and 5400 USD, 219 (16.6%) between 5400 and 8000 USD, and 137 (10.4%) between 8100 and 10,800 USD. An additional 101 participants (7.7%) reported earning over above 10,800 USD, and 147 (11.1%) chose not to disclose their income. Over 89% of the participants resided in one of the following five countries: the United States (281 participants; 21.3%), Israel (255; 19.3%), Italy (247; 18.7%), the United Kingdom (226; 17.1%), and Russia (166; 12.6%). The remaining 143 participants (10.8%) were distributed across 29 other countries. Of the participants, 237 (18.0%) had previously been or were currently diagnosed with cancer.

**Figure 2:**
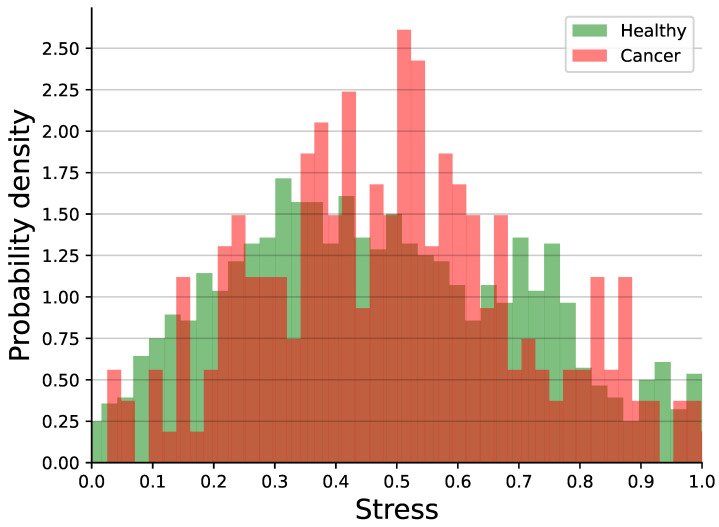
Histogram of the stress level of the participants divided into those who had cancer and those who did not.

Figure 2 shows the histogram of the participants’ stress levels, divided into participants who had cancer and those who did not. Initially, using the Shapiro–Wilk test [114], we tested whether the data was normally distributed, finding that stress levels for both healthy individuals and individuals with cancer were not normally distributed with p=0.008 and p=0.023, respectively. Using a Mann–Whitney U test, we showed there was no statistical difference between the two distributions with p=0.108.

Next, we computed the univariate statistical relationship between the variables with stress levels and the occurrence of cancer. Because the stress-related variables were not normally distributed, univariate associations involving stress were evaluated using the Spearman rank correlation coefficient, whereas associations involving cancer occurrence were evaluated using the $\chi^{2}$ test. Starting with the stress-related variables, the Spearman rank correlation analysis showed that cancer treatment had a relatively high positive association with stress (ρ=0.34, p<0.01). In addition, individuals with a higher number of cancer cases in the family tended to report higher stress levels (ρ=0.19, p<0.01). Furthermore, stress frequency was moderately positively associated with stress levels (ρ=0.55, p<0.01), followed by the belief that stress affects one’s health (ρ=0.30, p<0.05). In a similar manner, self-evaluation of coping with stress showed a relatively small negative association with self-reported stress levels (ρ=−0.14, p<0.05). Among the demographic variables, age, number of children, and education level showed small positive associations with stress (ρ=0.03, p<0.05; ρ=0.10, p<0.01; and ρ=0.06, p<0.05, respectively). The remaining variables did not show statistically significant associations with stress.

Table 1 presents the results of a logistic regression analysis examining factors associated with the occurrence of cancer. Several variables demonstrated statistically significant associations with the odds of a cancer occurrence. Specifically, older age β=0.090, p=0.002, a family history of cancer β=0.128, p=0.003, higher number of family members with cancer β=0.104, p=0.019, higher stress frequency β=0.031, p=0.045, greater perceived health impact of stress β=0.062, p=0.047, and higher general stress levels β=0.091, p=0.034 were all positively associated with increased odds of cancer occurrence. While the estimate for higher education level was positive β=0.017, it did not reach statistical significance (p=0.094). Similarly, gender, marital status, number of children, income, household size, stress-coping ability, sleep issues, appetite loss, and fatigue did not show statistically significant associations with cancer occurrence in this model. Variables that did not reach statistical significance, including stress-coping ability, were nevertheless retained in the multivariable models as prespecified covariates for consistency and adjustment; therefore, their inclusion in the causal path diagram reflects model specification rather than evidence of a statistically significant independent effect. In this context, collinearity diagnostics indicated that multicollinearity was not a concern in the logistic regression model, as all independent variables had VIF values below the commonly accepted threshold (2.7–4.8 < 5). In addition, the Hosmer–Lemeshow goodness-of-fit test was non-significant ($\chi^{2}=11.03$, df=8, p=0.2), suggesting an adequate fit between the model predictions and the observed outcomes.

### 4.2. Machine Learning Results

Table 2 presents the performance metrics of the ML models used for predicting cancer occurrence based on socio-demographic, stress-related, and immune suppression factors. The XGBoost-based model achieved the highest performance, with an accuracy of 0.73 and an F1 score of 0.68. These values imply close-to-optimal predictive capabilities on the proposed data (which have moderate predictive performance), as XGBoost is considered the SOTA on tabular data [92]. The DT-based model performed moderately well, but significantly worse compared to XGBoost, with an accuracy of 0.64 and an F1-score of 0.60. The KNN classifier was tested on different feature sets. When using all variables, the KNN model achieved an accuracy of 0.68 and an F1 score of 0.66, showing better performance compared to the DT model and worse performance compared to the XGBoost model. When trained on only socio-demographic variables, stress-related variables, or immune suppression factors, its performance marginally declined. The latter two were just slightly better than a random guess (which will produce a 0.5 score).

The XGBoost model’s superior accuracy highlights its ability to effectively capture complex interactions within the data and suggests that the interactions among socio-demographic, stress-related, and immune suppression variables are intricate and nonlinear. The Decision Tree (DT) model, a simpler, hierarchical splits, global model that offers the advantage of being interpretable, demonstrated lower performance compared to XGBoost. This performance may indicate that the relationships in the data are not easily captured through straightforward hierarchical splits. The k-Nearest Neighbors (KNNs) classifier, which showed moderate performance when using all variables, provides an interesting contrast. Its performance superiority over DT yet inferiority to XGBoost implies that while the KNN model takes advantage of the proximity-based relationships in the data, it may struggle with scalability and dimensionality compared to XGBoost. The decline in KNN’s performance when restricted to subsets of variables (socio-demographic, stress-related, or immune suppression) may indicate that cancer occurrence is likely influenced by a combination of factors rather than isolated variable groups. The relatively better performance for the socio-demographic variables subset may indicate, however, that cancer occurrence dynamics are directly associated with socio-economic properties but are not directly associated with stress levels and immune suppression.

**Figure 3:**
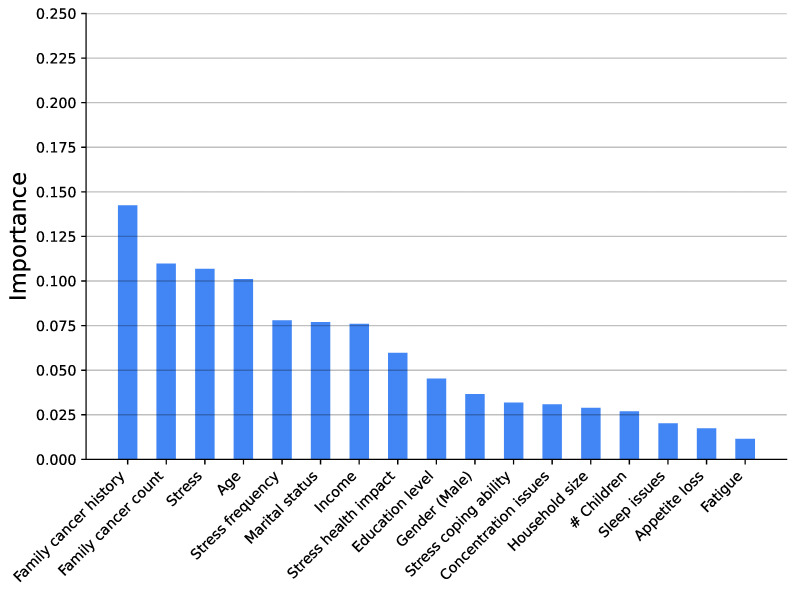
Feature importance distribution for the XGBoost model predicting cancer occurrence (# is number of).

**Figure 4:**
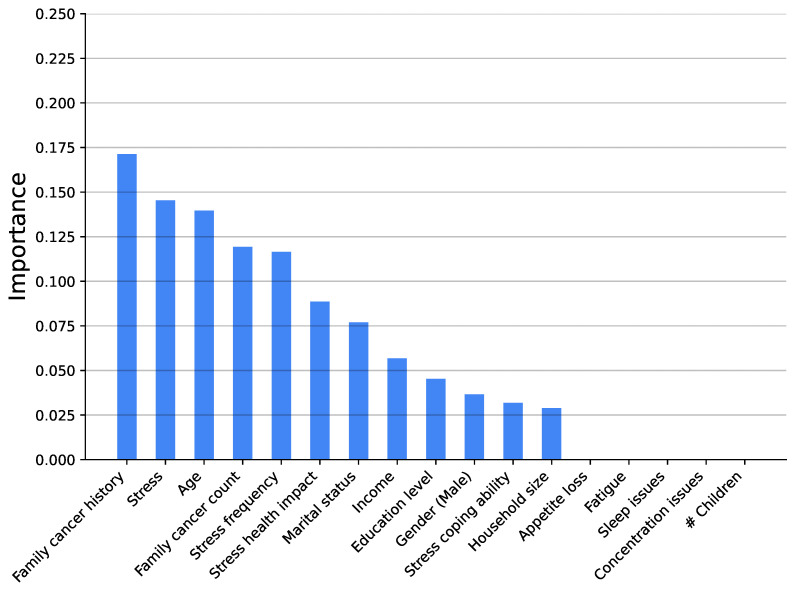
Feature importance distribution for the Decision Tree model predicting cancer occurrence (# is number of).

**Figure 5:**
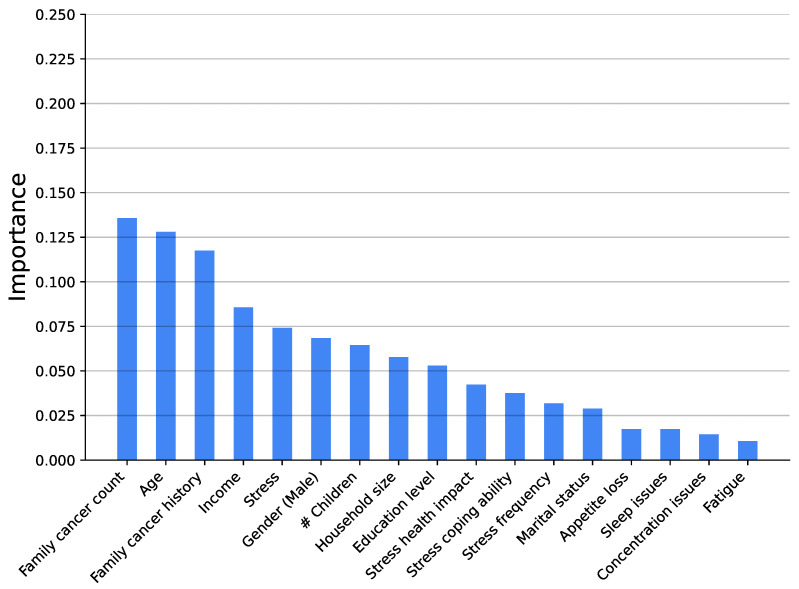
Feature importance distribution for the KNN model trained on all variables (# is number of).

**Figure 6:**
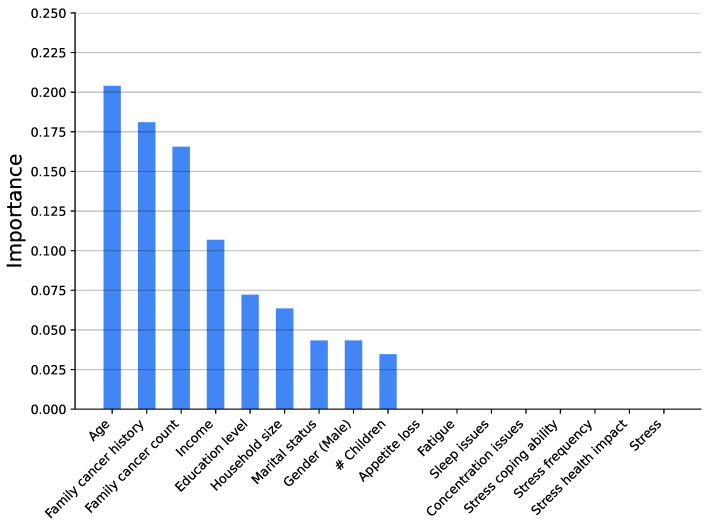
Feature importance distribution for the KNN model trained only on socio-demographic variables (# is number of).

**Figure 7:**
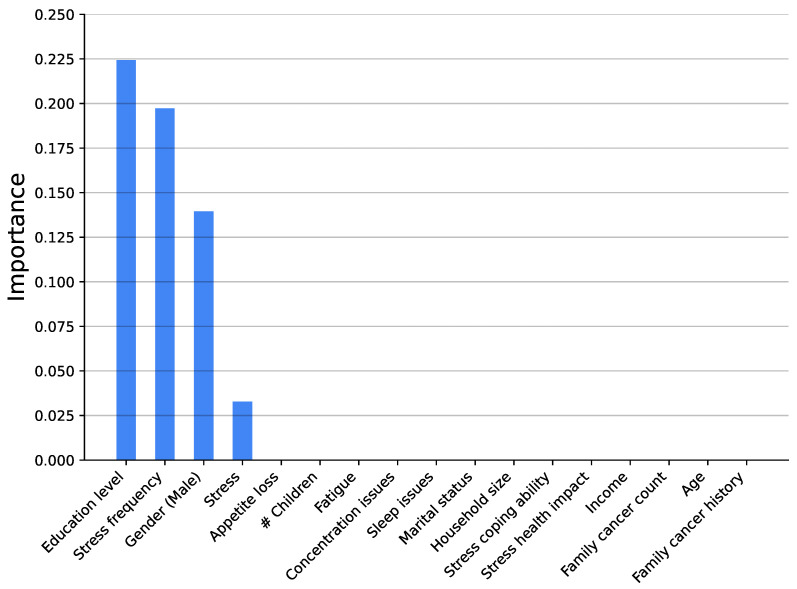
Feature importance distribution for the KNN model trained only on stress-related variables (# is number of).

**Figure 8:**
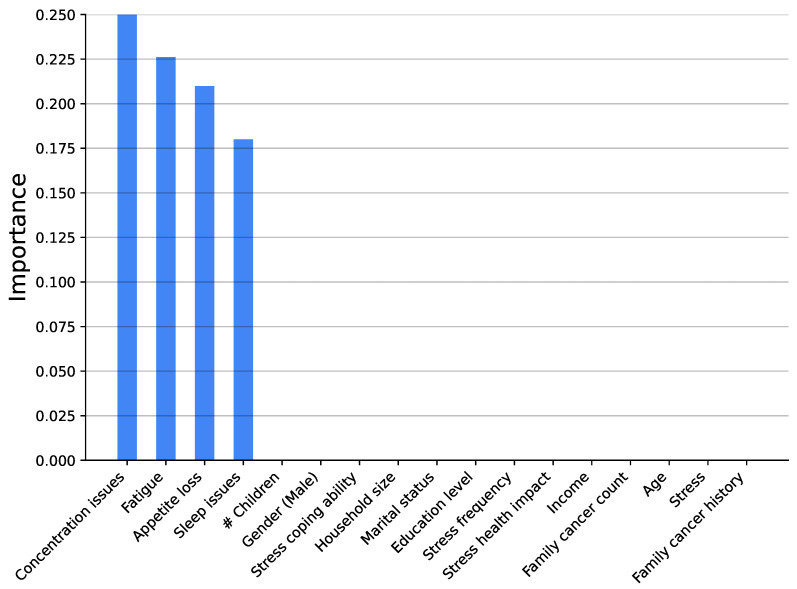
Feature importance distribution for the KNN model trained only on immune suppression variables (# is number of).

Figure 3, Figure 4, Figure 5, Figure 6, Figure 7 and Figure 8 show the variable’s importance analysis for the six obtained ML models. For all the models that include family cancer history, it is the most important variable. Age is also found to be important across the models, being the second most important variable in KNN model as well as fourth and third for the XGBoost and DT models, respectively. For the (global) tree-based models, XGBoost and DT, the level of stress is the third and second most important variable, respectively, while for the (local) similarity-based KNN model, stress is the fifth most important variable. For further exploration of the model’s explainability, we provide SHAP analysis [115,116].

### 4.3. Rubin-Model Effect-Estimation Results

For the Rubin-model analysis, we adopted a two-phase strategy: first, we estimated direct assumption-dependent effects between all model variables and cancer occurrence; second, we examined a prespecified pathway graph based on the literature. This approach allows us to obtain a simple yet straightforward causal relationship on the one hand and a more complex and theory-driven one on the other hand.

**Figure 9:**
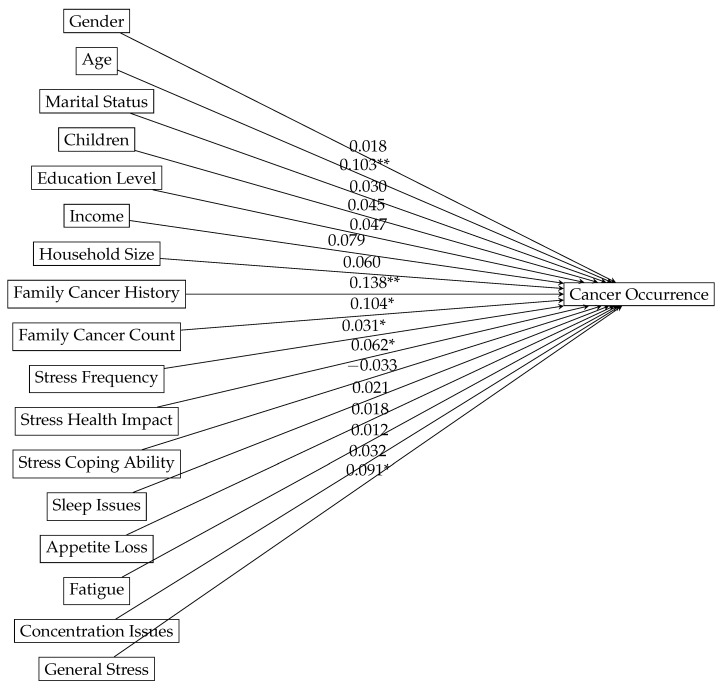
Path diagram of the direct causal model between the features and cancer occurrence. Standardized path coefficients are shown for all prespecified covariates, while significance levels are indicated only for statistically significant paths (* *p* < 0.05, ** *p* < 0.01).

Figure 9 presents the direct effect-estimation model for cancer occurrence, assuming direct associations of all variables with the outcome under the Rubin-model assumptions. Notably, age (0.103, p<0.01) and family cancer history (0.138, p<0.01) exhibit the strongest associations with cancer occurrence, highlighting their critical role in risk assessment. Other significant contributors include family cancer count (0.104, p<0.05), stress frequency (0.031, p<0.05), stress health impact (0.062, p<0.05), and stress levels (0.091, p<0.05), underscoring the interplay between genetic predisposition and psychological stress in cancer risk. These results suggest that while hereditary factors remain dominant, psychosocial stressors may also be meaningfully associated with cancer occurrence under the modeling assumptions. At the same time, because the data are cross-sectional, the temporal direction of this association cannot be established. In particular, elevated stress levels may reflect the consequences of cancer diagnosis, treatment, or related health concerns rather than a factor that preceded cancer occurrence, and reverse causation therefore remains a plausible explanation for part of the observed association.

**Figure 10:**
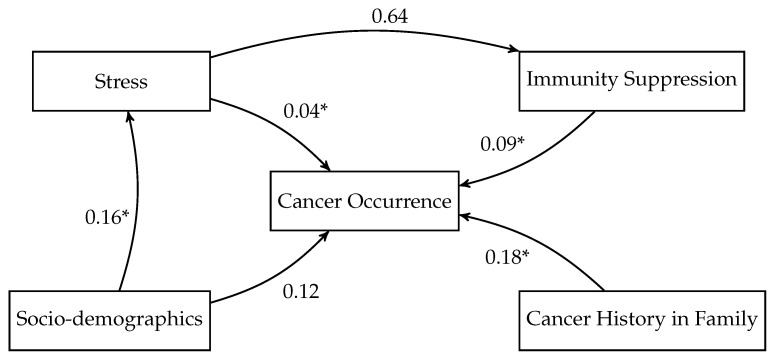
Path diagram of hypothesized relationships influencing cancer occurrence. Standardized path coefficients and significance levels are indicated (* *p* < 0.05).

Figure 10 presents a hypothesized model for the relationships between stress and cancer occurrence while controlling for potential confounding factors—socio-demographics, immune suppression, and cancer history in the family. Specifically, the model posits that stress may directly influence cancer occurrence (β=0.04,p<0.05), as well as indirectly through immunity suppression (β=0.64), which in turn impacts cancer occurrence (β=0.09, p<0.05). The model further incorporates cancer history in family (β=0.18, p<0.05) and socio-demographics as direct predictors of cancer occurrence, allowing us to assess their independent contributions. Finally, socio-demographics are also hypothesized to influence stress levels (β=0.16, p<0.05), acknowledging the potential role of social factors in shaping stress responses. However, although this pathway model was motivated by the prior literature, the estimated indirect effect of stress on cancer occurrence through immune suppression was not statistically significant in the present data. Therefore, the model should be interpreted as testing a hypothesized mediation structure rather than as confirming an immune-mediated mechanism. In addition, the presence of associations among individual component paths does not by itself imply a statistically significant indirect effect.

## 5. Discussion and Conclusions

Our study explores the contribution of chronic stress and immune suppression to cancer occurrence. Insights from this association have significant implications for precision medicine strategies aimed at mitigating stress-induced changes in immune function [5,18]. Quantifying and understanding this association may help in developing interventions to reduce cancer risk and improve treatment outcomes [6].

Our results offer a nuanced understanding of cancer risk factors. They integrate survey data from four key categories of variables that were hypothesized as related to cancer occurrence: stress, socio-demographic factors, family cancer history, and immune suppression.

Our machine learning models effectively capture the interactions between variables. These findings reinforce the significance of stress in cancer prediction, and highlight the potential of machine learning in capturing and modeling cancer risk [12,13,17,103]. Specifically, the combination and comparison of different ML models is demonstrated as effective, where the better prediction accuracy of XGBoost was combined with the explainability of KNNs. Importantly, these predictive results should not be interpreted as evidence that the present model is ready for direct clinical deployment. The observed performance is more appropriately viewed as indicating that the collected variables, including stress-related measures, contain non-random predictive information relevant to cancer occurrence. From a public health perspective, this is useful because it supports the value of incorporating psychosocial stress indicators into future epidemiological and clinical studies of cancer risk. At the same time, the model is intended to motivate further validation and refinement, rather than to replace established risk assessment strategies in clinical practice. Notably, when compared with a parsimonious logistic regression baseline based on age and family cancer history, the XGBoost model showed higher predictive performance, suggesting that the inclusion of additional stress-related and socio-demographic variables provides incremental predictive value.

Our traditional statistical methods corroborate these insights and indicate significant correlations between stress variables and cancer risk [10,49].

However, our Rubin-model analysis goes beyond simple bivariate correlation by providing a structured, assumption-based framework for evaluating how these factors may relate to cancer occurrence. The most important findings of the study were the identified structured associations and assumption-dependent estimated effects between both variable categories (Figure 10), individual variables (Figure 9), and cancer occurrence. Age and family cancer history (Figure 9) are revealed as dominant factors in cancer risk, corroborating the importance of hereditary influences [2,67]. Psychosocial stressors, including stress frequency, health impact, and stress levels, also play a significant role, as suggested previously [7,18]. Our model (Figure 10) suggests that both stress and immunity suppression directly influence cancer. The indirect effect of stress on cancer occurrence through immune suppression was not significant according to our causal model [32,33]. These findings add depth to existing research by quantifying these relationships and their interconnections. The significant associations between stress frequency, stress health impact, and cancer occurrence suggest that individuals who perceive stress as significantly affecting their health and experience frequent stress are more likely to report a history of cancer [7,22]. This could be due to stress-related behavioral changes and physiological effects impacting cancer risk [32], but it may also partly reflect reverse causation, whereby cancer diagnosis, treatment burden, or broader health concerns elevate perceived stress levels [32]. The significant correlation with age was previously associated with immunosenescence, which exhibits similarities to stress-induced immune alterations. Elderly individuals frequently display elevated cortisol levels and hypothalamic–pituitary–adrenal axis activation, potentially exacerbating stress-related immune dysfunction [49,117]. Older age may cause cancer risk due to cumulative exposure to environmental risk factors over time and the natural accumulation of genetic mutations [2,3]. These insights are valuable additions to the existing body of epidemiological studies that displays contradicting evidence of stress correlation to cancer risk [10,25,26].

The findings suggest actionable insights into the mitigation of cancer risk. The causal effects of stress variables reflect chronic stress exposure, the way individuals perceive and experience stress, and unhealthy coping mechanisms such as poor diet, lack of exercise, and substance use [50]. This suggests that interventions directly targeting stress management might be effective as cancer prevention strategies.

Our methodology entails several limitations. The reliance on self-reported data introduces potential biases, affecting the precision of stress and health assessments [27]. Online self-recruited participants may limit the generalizability of the findings to populations that do not have internet access or are underrepresented—individuals with low socio-economic status or those in rural areas—in online surveys [61]. Specific questions—history of cancer diagnosis, stress-related experiences—are vulnerable to recall bias, where participants might inaccurately recall past events or experiences [10]. The survey variables of stress and immune suppression may oversimplify the nuanced physiological and psychological dimensions of chronic stress and immune function, compared to objective biomarkers such as stress-related hormones like cortisol or immune-related cytokines [18,31,33]. The study’s cross-sectional design limits the ability to draw definitive causal conclusions [10], and longitudinal data is needed [29]. Moreover, while causal modeling can control for observed confounders, unmeasured variables (e.g., smoking or alcohol consumption) may still confound the observed relationships. This is particularly relevant to stress and cancer risk, as both may be influenced by hidden shared factors [10,28]. An additional limitation stems from the voluntary online recruitment strategy itself. Because participants were recruited through open online dissemination, the study is subject to internet access bias and self-selection bias, and the resulting sample may overrepresent individuals who are more digitally connected, more health-aware, or more motivated to respond to surveys. Furthermore, as recruitment did not rely on a closed sampling frame, the denominator of individuals exposed to the invitation is unknown; accordingly, the sampling frame and response rate could not be reported. This limitation is important when interpreting the external validity of the findings, as the observed associations may not generalize equally to populations that are underrepresented in online surveys. Social desirability bias may have additionally influenced the reporting of stress-related experiences and health perceptions despite the anonymous format. Future studies would benefit from combining online recruitment with structured probability-based or clinic-based sampling strategies, and from reporting sampling frame coverage and participation rates whenever feasible.

Taken together, our study highlights the multidimensional nature of cancer occurrence [50], and the combined associations of genetic predisposition, stress perception, and socio-demographic factors, while leaving the hypothesized immune-mediated pathway unresolved. Longitudinal studies should explore these relationships over time to better understand their dynamics and potential for intervention. Our study shows the way in this context, as it reinforces the need for approaches that integrate diverse data types to capture the full spectrum of influences on cancer occurrence [11]. Our findings highlight the benefit of integrating machine learning and causal models in the discovery of patterns that traditional and simpler models might miss [12]. These patterns provide greater insight into complex health datasets of cancer, and enhance the ability to develop predictive models for cancer occurrence [11,13]. By identifying modifiable stress-related factors and quantifying their impact on cancer occurrence, these models can inform targeted prevention strategies and enhance early screening protocols tailored to high-risk individuals [7,24,29]. Addressing psychological stress through interventions such as stress reduction techniques, lifestyle modification programs, and psycho-oncological support has been shown to effectively reduce cancer risk when integrated into broader prevention frameworks [18,22]. Evidence from predictive models could guide the development of public health interventions aimed at reducing the global cancer burden.

Beyond these immediate extensions, the combined ML and causal framework used in this study also suggests several more advanced directions for future work. First, longitudinal multimodal studies could integrate repeated self-reports with biological measurements, electronic health records, wearable-device data, and digital phenotyping in order to model how stress-related processes evolve over time and whether they precede measurable immune and oncological changes. Second, individualized causal estimation methods could be employed to move from average treatment effects toward patient-specific counterfactual risk profiles, thereby identifying subpopulations for whom stress-related pathways may be especially influential. Third, causal discovery and structure-learning approaches may help refine the hypothesized relationships between psychosocial, biological, and socio-demographic variables beyond the prespecified causal graph used here. Finally, future predictive models could explicitly compare simple clinical baselines with hybrid explainable ML systems, combining strong predictive performance with transparent decision rules that are more suitable for translation into public health and precision-medicine settings.

## Figures and Tables

**Table 1:** Logistic regression results for cancer occurrence. Notably, marital status and level of education are treated as categorical variables, with the reference category indicated in parentheses.

Q-Index	Parameter	Estimate	*p*-Value	95% CI
1	Gender (Male)	−0.038	0.409	[−0.128, 0.052]
2	Age	0.090	0.002	[0.031, 0.149]
3	Marital Status (Ref: Single)	−0.080	0.151	[−0.189, 0.029]
4	Children	−0.028	0.277	[−0.077, 0.021]
5	Education Level (Ref: Low)	0.017	0.094	[−0.003, 0.037]
6	Income	0.069	0.149	[−0.025, 0.163]
7	Household Size	0.060	0.315	[−0.058, 0.178]
8	Family Cancer History (Yes)	0.128	0.003	[0.042, 0.214]
9	Family Cancer Count	0.104	0.019	[0.017, 0.191]
10	Stress Frequency	0.031	0.045	[0.001, 0.061]
11	Stress Health Impact	0.062	0.047	[0.001, 0.123]
12	Stress Coping Ability	−0.033	0.067	[−0.069, 0.003]
13	Sleep Issues	0.021	0.483	[−0.040, 0.082]
14	Appetite Loss	−0.018	0.253	[−0.050, 0.014]
15	Fatigue	0.012	0.070	[−0.001, 0.025]
16	Concentration Issues	0.032	0.186	[−0.048, 0.112]
17	Stress (General)	0.091	0.034	[0.007, 0.175]

**Table 2:** Performance metrics for different models.

Model	Accuracy	F1 Score	Recall	Precision
XGBoost	0.73	0.68	0.64	0.72
DT	0.64	0.60	0.57	0.63
KNN (all variables)	0.68	0.66	0.63	0.69
KNN (only socio-demographic)	0.61	0.59	0.53	0.66
KNN (only stress)	0.54	0.53	0.55	0.51
KNN (only immune suppression)	0.56	0.55	0.56	0.53

## Data Availability

Data available on request due to restrictions.

## Appendix A. Questionnaire

The online study consisted of the following questions and possible answers as closed-form questions.

- “Country”: Open-ended.
- “Gender”: Male, Female, Prefer not to say.
- “Age”: 18–120.
- “What is your marital status?”: Single, in a relationship, married, divorced, widow/er.
- “How many children do you have?”: 0, 1, 2, 3, 4, 5+.
- “Education”: Did not complete high school, high-school, Bachelor’s, Master’s degree, PhD/M.D.
- “Household Monthly Income (before taxes and deductions) from all sources”: up to 2150 USD monthly, 2150–2700 USD monthly, 2700–3250 USD monthly, 3250–5400 USD monthly, 5400–8100 USD monthly, 8100–10,800 USD monthly, 10,800 USD monthly, would prefer not to answer.
- “How many people live with you in the same house?”: 1–15.
- “Have you ever been diagnosed with cancer?”: Yes, No.
- “If yes, what type of cancer were you diagnosed with?”: Open-ended.
- “If yes, when were you diagnosed?”: Less than 1 year ago, 1–3 years ago, 3–5 years ago, Over 5 years ago.
- “Are you currently undergoing cancer treatment?”: Yes, No.
- “Have any of your immediate family members been diagnosed with cancer?”: Yes, No.
- “If yes, how many family members have been diagnosed with cancer?”: 1, 2, 3, 4, 5+.
- “If yes, what is your relationship to the closest diagnosed family members?”: Parent, Sibling, Child, Other (open-ended question).
- “If yes, what type of cancer was your closest family member diagnosed with?”: Open-ended.
- “How often do you feel stressed in your daily life?”: Never, Rarely, Sometimes, Often, All the time.
- “Do you believe stress affects your physical health?”: Strongly agree, Agree, Neutral, Disagree, Strongly disagree.
- “How well do you cope with stress?”: Very well, Well, Average, Poorly, Very poorly.
- “In the past month, how often have you experienced difficulty sleeping?”: Never, Rarely, Sometimes, Often, All the time.
- “In the past month, how often have you experienced loss of appetite?”: Never, Rarely, Sometimes, Often, All the time.
- “In the past month, how often have you experienced fatigue?”: Never, Rarely, Sometimes, Often, All the time.
- “In the past month, how often have you experienced difficulty concentrating?”: Never, Rarely, Sometimes, Often, All the time.
- “How much do you feel that stress has impacted your mental health in the past year?”: Not at all, A little, Moderately, Significantly, Extremely.
